# An Isotope-Induced Polarity Bias in Electrospray Ionization Mass Spectrometry

**DOI:** 10.1021/acsmeasuresciau.6c00134

**Published:** 2026-07-14

**Authors:** Qi Ying Neo, Henry T. P. Annecke, Irena Papageorgiou, Axel Leppert, Katja Petzold, Kevin Pagel, Ali Miserez, Erik G. Marklund, Nicklas Österlund, Michael Landreh

**Affiliations:** † Department of Microbiology, Tumor and Cell Biology, 27106 Karolinska Institutet, Solna 171 65, Sweden; ‡ Center for Sustainable Materials (SusMat), School of Materials Science and Engineering, 54761 Nanyang Technological University, Singapore 639798, Singapore; § Department of Medical Biochemistry and Microbiology & Science for Life Laboratory & Center of Excellence for the Chemical Mechanisms of Life, 8097 Uppsala University, Uppsala Biomedical Centre, Uppsala 752 37, Sweden; ∥ Department of Medical Biochemistry and Biophysics, 27106 Karolinska Institutet, Stockholm 171 65, Sweden; ⊥ Department for Cell and Molecular Biology, 8097 Uppsala University, Uppsala 751 24, Sweden; # Institute of Chemistry and Biochemistry, Freie Universität Berlin, Berlin 14195, Germany; ¶ Department of Molecular Physics, Fritz Haber Institute of the Max Planck Society, Berlin 14195, Germany; ∇ Department of Chemistry for Life Sciences, 8097 Uppsala University, Uppsala 751 23, Sweden

**Keywords:** electrospray ionization, native mass spectrometry, heavy water, negative ion mode, polarity-dependent ionization, protein signal suppression

## Abstract

Heavy water (D_2_O) is commonly used as a solvent additive in electrospray ionization-mass spectrometry (ESI-MS) to probe biomolecular interactions under the assumption that isotopic substitution does not significantly affect the ionization process. However, D_2_O differs measurably from H_2_O in several physicochemical properties, which can influence ionization across biomolecular classes. Here, we systematically investigate how D_2_O influences native ESI across biomolecular classes and show that D_2_O introduces a pronounced, polarity-dependent detection bias which can suppress the signal of proteins and peptides in negative ion mode relative to other classes of biomolecules. Protein signal suppression is mitigated by reducing primary ESI droplet size or adding volatile ammonium salts, implicating altered droplet evolution and salt retention in D_2_O. Its magnitude is strongly dependent on analyte acidity: basic and moderately acidic proteins are most affected, whereas extremely negatively charged proteins, RNA, and polysaccharides remain largely unaffected. Signal loss also correlates with extensive sodium adduction and peak splitting rather than impaired charging. Exploiting this selectivity, we demonstrate controlled switching between peptide and RNA detection in a coacervate-based cellular delivery system. These findings reveal an unexpected solvent-dependent bias in negative-mode native MS measurements which can be exploited for experimental design.

## Graphical abstract

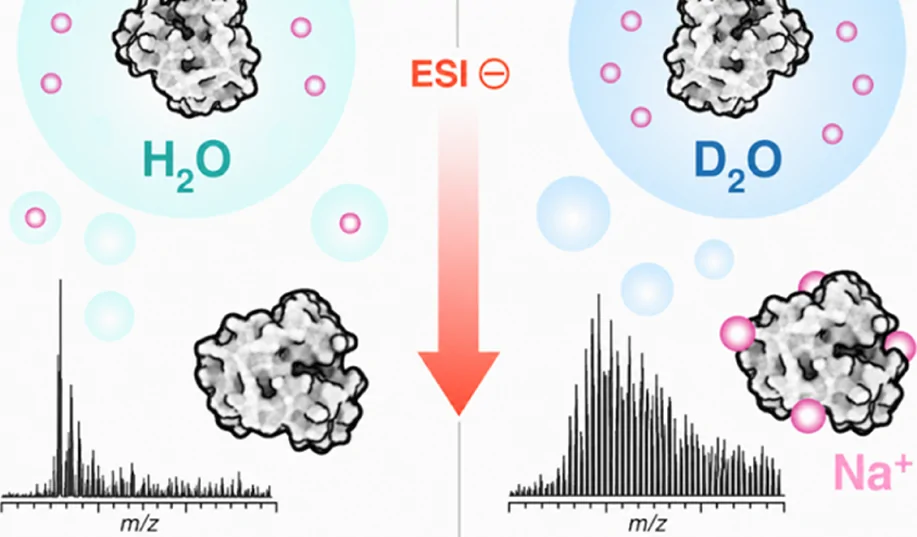

## Introduction

Mass spectrometry (MS) is a powerful analytical technique that enables sensitive and selective identification and quantification of compounds by measuring the mass-to-charge (*m*/*z*) ratios of gaseous ions. The development of electrospray ionization (ESI) allows the characterization of biomolecular assemblies while preserving noncovalent interactions and folded biomolecular structures.^1,2^ ESI transfers intact biomolecules from aqueous solution into the gas phase of a mass spectrometer via the formation and desolvation of charged droplets.^3,4^ During the ionization process, droplets undergo rapid solvent evaporation, increasing charge density and repeated cycles of evaporation and Coulombic fission, leading to the production of multiply charged ions. The efficiency of this process depends sensitively on solvent parameters such as droplet viscosity and surface tension.^5–7^ Consequently, changes in solvent composition markedly influence ion formation and desolvation behavior, while also affecting biomolecular structure in solution.^8–11^

Heavy water i.e. deuterium oxide (D_2_O) is used as a solvent in techniques such as nuclear magnetic resonance (NMR), neutron scattering and infrared spectroscopy.^12^ Compared to protium water (H_2_O), D_2_O forms stronger hydrogen bonds and more structured hydrogen-bonding networks, resulting in altered hydration, increased solvent viscosity, and modified acid–base properties.^12,13^ These changes generally favor more compact biomolecular conformations in solution, with strengthened intramolecular interactions and reduced conformational flexibility.

Recently, ESI-MS has been used to probe the effects of D_2_O on the stability and interactions of intact proteins. In solution, D_2_O stabilizes small monomeric proteins like cytochrome *c* and lysozyme, but this effect is not reflected in their gas-phase unfolding behavior.^14^ Desolvated deuterated and undeuterated proteins exhibit indistinguishable gas-phase unfolding, indicating that isotope substitution itself does not alter their kinetic stability. Instead, changes in stability arise from solvent–solvent and solvent–protein interactions.^14–16^ However, solvent effects can still be probed with native MS. Variants of tetrameric transthyretin exhibit pronounced differences in gas-phase stability when electrosprayed from H_2_O versus D_2_O. These differences can be attributed to solvent-dependent changes at interfacial hydration that are preserved during ESI.^17^ Similarly, D_2_O affects the cooperativity of ligand binding, further supporting the concept that native MS can probe hydration effects.^18^

Such solvent-dependent effects raise the question of whether D_2_O may also influence droplet evolution and desolvation during ESI. Given the established importance of solvent properties such as p*K*_a_, viscosity, and surface tension for ESI,^12^ we asked whether the distinct physicochemical properties of H_2_O and D_2_O can influence desolvation and ion formation during ESI. To address this question, we examined the ionization behavior of polypeptides, polysaccharides, and polynucleotides electrosprayed from H_2_O and D_2_O solutions in both positive and negative ion modes. We find that protein ionization from D_2_O is selectively suppressed in negative ion mode and is accompanied by extensive adduct formation. These effects arise from solvent-dependent changes in droplet evolution and desolvation efficiency. Beyond providing mechanistic insight into solvent effects in ESI, our findings offer a strategy for selectively suppressing protein signals in complex biomolecular mixtures, enabling enhanced detection of nonprotein components by native MS.

## Results

### D_2_O Suppresses Protein Signals in Negative Ion Mode

To identify potential effects of D_2_O on the ESI process, we selected cytochrome c, a well-studied protein that undergoes D_2_O-induced conformational shifts in solution but not in the gas phase.^14,19^ As the first step, we analyzed cytochrome *c* in pure H_2_O/D_2_O, where the D_2_O content of the ESI solution was increased stepwise from 0 to 90%. Mass spectra were recorded in positive ion mode, then the polarity of the instrument was flipped and spectra were recorded in negative ion mode with no further instrument tuning (Figure 1A). In positive ion mode, we observed a D_2_O content-dependent increase in protein mass, as expected from deuterium incorporation into the backbone and side-chains (Figures 1B, S1A). In negative ion mode in the absence of D_2_O, the protein has a lower number of charges than in positive ion mode and up to four high-intensity sodium adduct peaks (Figure 1B). Strikingly, upon adding 10% D_2_O to the ESI solution, we observed a further increase in the number of adducts. This trend was even more pronounced in 25% D_2_O where we detected up to 9 high-intensity sodium adduct peaks. In 90% D_2_O, the protein was not detectable in negative ion mode.

**Figure 1:**
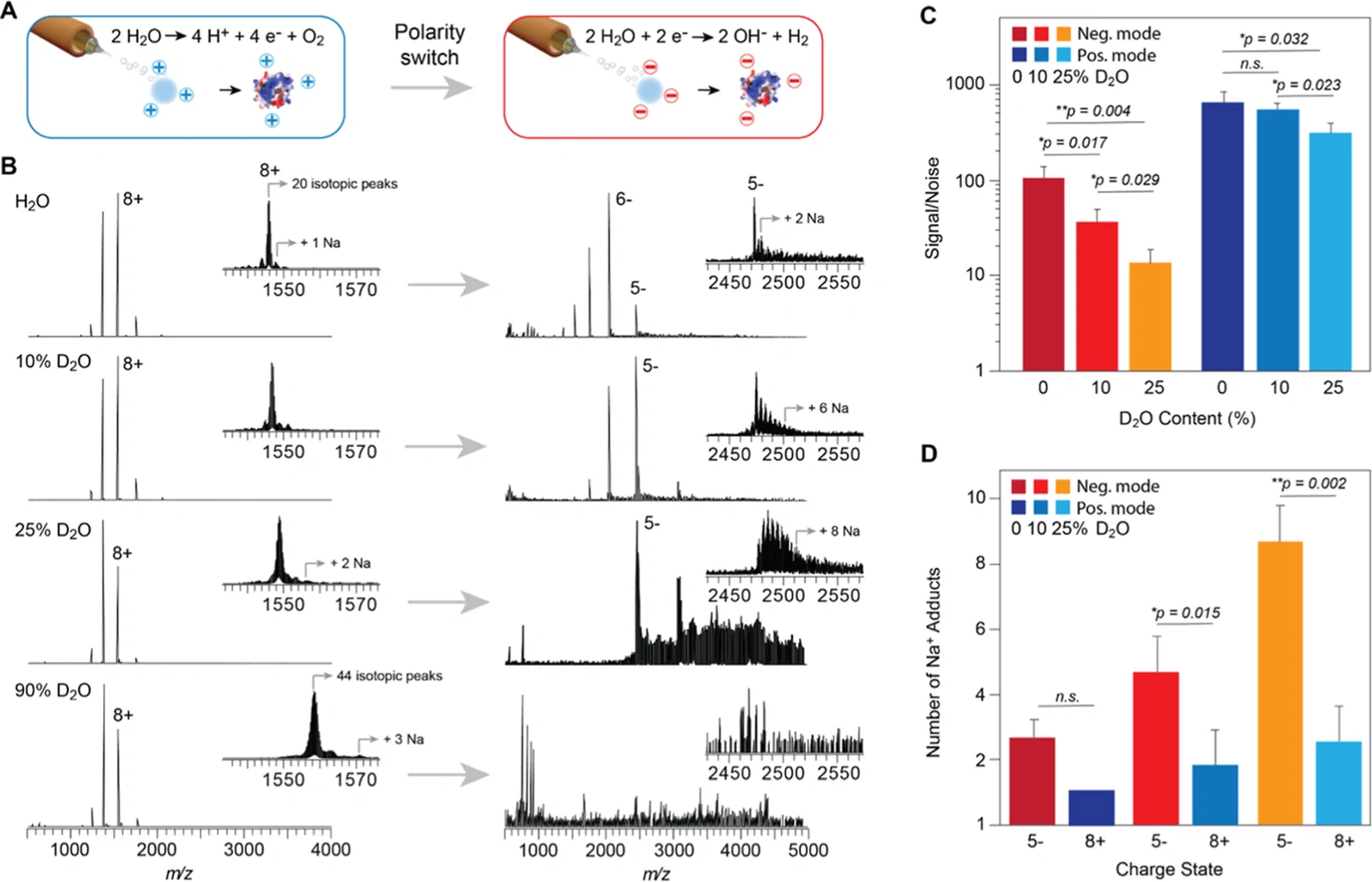
D_2_O causes cytochrome *c* protein signal suppression and sodium adduction in negative ion mode. (A) Experimental setup. A nanospray capillary is loaded with cytochrome *c* with 0–90% D_2_O and spectra are recorded in positive ion mode, before switching the instrument polarity and recording in negative mode with no further tuning. (B) In positive ion mode, no change in sodium adduct formation is observed. The 8+ charge state with sodium adduct is shown as an insert. In negative ion mode, the number of sodium adducts increases with increasing D_2_O content. The presence of 90% D_2_O leads to near-complete protein signal suppression. The 5- charge state is shown as an inset with the number of sodium adducts indicated. (C) Quantification of the S/N for the largest peak in each spectrum. The addition of 25% D2O leads to a 50% reduction in S/N in positive ion mode, and a reduction by 1 order of magnitude in negative ion mode. (D) Quantification of the maximum number of clearly distinguishable sodium adducts. Data in panels C and D are shown as average and standard deviation (*n* = 4). Significances (Student’s *t*-test, two-tailed distribution with equal variance) are indicated above the bars.

We then sought to quantify the effect of D_2_O on spectral quality by plotting the average signal-to-noise (S/N) for the main charge state (highest peak) in each spectrum (Figure 1C). We found that 25% D_2_O reduced S/N by ca. 50% in positive and nearly 10-fold in negative ion mode. Since deuterium incorporation causes broadening of the isotope distribution of the protein signal, a reduction in S/N by D_2_O is expected. The 50% reduction in S/N observed in positive mode is in good agreement with broadening of the isotope envelope from approximately 20 to 44 isotopic peaks (Figure 1B). Importantly, no notable differences in deuterium incorporation were found between both ion modes (Figure S1B), indicating that S/N reduction does not stem purely from deuterium labeling. Quantification of the number of sodium adducts confirms the significant adduct increase in negative ion mode by D_2_O (Figure 1D). However, the low *m*/*z* regions of the spectra recorded in negative ion mode show no traces of NaCl clusters, suggesting no significant salt contamination (Figure S1B).

### Protein Signal Suppression is Partially Due to D_2_O-Mediated Sodium Adduction

To test whether the observed adduct formation and signal degradation arise from trace salt contamination in the D_2_O solvent, we conducted control experiments in which cytochrome *c* was electrosprayed from H_2_O supplemented with NaCl at concentrations of 1 mM and 10 mM (Figure S2A). Under these conditions, well-resolved charge-state distributions were obtained in both ionization modes, in contrast to the spectra acquired from D_2_O-containing samples, despite the intentionally elevated salt levels. As expected, sodium adduct formation increased with NaCl concentration. However, the overall spectral quality remained relatively high and did not resemble the extensive adducting and protein signal suppression observed for D_2_O-containing samples of cytochrome *c* in negative mode. These results indicate that higher amounts of trace salt impurities in the D_2_O solvent cannot account for the poor spectral quality and that D_2_O itself adversely affects the electrospray ionization process in negative ion mode.

Having ruled out salt contamination, we next considered the pronounced increase in sodium adduct formation as an indicator of altered electrospray droplet formation and desolvation behavior in D_2_O. To test this possibility, we subjected the protein to in-source fragmentation in the presence of 50% D_2_O. Briefly, the protein ions undergo high-energy collisions with gas molecules in the ion source, which can dissociate salt adducts.^20^ We find that in-source fragmentation partially mitigates protein signal suppression, suggesting that it is caused by extensive adduct formation (Figure 2A). The significant increase in sodium adduct formation with D_2_O in negative ion mode indicates that the protein ions originate from large primary ESI droplets that carry a higher number of nonvolatile salt ions. To verify if this is the case, we performed negative mode MS of cytochrome *c* using nano-ESI capillaries with an approximate diameter of 1 μm, approximately 10 times smaller than the capillaries used in the earlier experiments,^21,22^ which consequently produce smaller primary ESI droplets. The production of smaller primary droplets implies that fewer rounds of evaporation and Coulombic fission are required to form smaller desolvated offspring droplets, addressing inefficient desolvation. In H_2_O, we observe an additional peak for the 6-charge state of cytochrome *c* in comparison to using the larger ESI tip (Figure 2B). In 87% D_2_O, the 5- and 6-charge state peaks remain well resolved. The number of sodium adducts was comparable to that observed in 25% D_2_O with the larger ESI tips. By pulling needles with larger tip openings we could see a progressive worsening of spectral quality in D_2_O, but not in H_2_O, indicating that negative mode ESI in D_2_O is more sensitive to the size of the primary ESI droplets (Figure S2B). Sodiation at high D_2_O content could be further suppressed by addition of volatile ammonium acetate salt (Figure S2C), presumably by outcompeting binding to nonvolatile salts.

**Figure 2:**
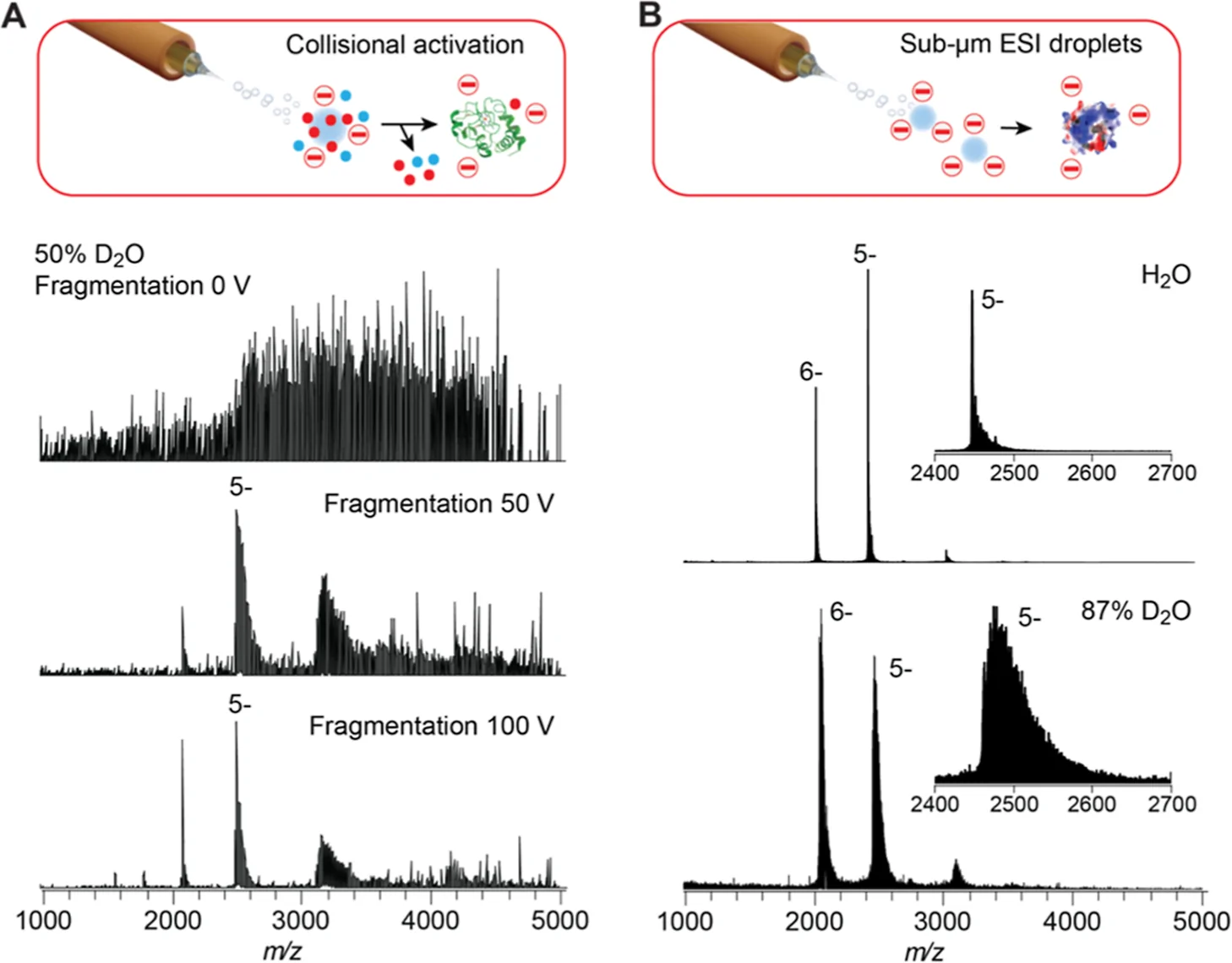
Protein signal suppression is partially mitigated by reducing sodium adduction. (A) Increasing the in-source fragmentation energy from 0 to 100 V improves cytochrome *c* detection in the presence of 50% D_2_O by stripping sodium adducts during desolvation. (B) The use of ESI capillaries with a 1 μm diameter to produce submicrometer droplets facilitates detection of cytochrome *c* in 87% D_2_O in negative ion mode. Sodium adduction is comparable to the levels seen with 25% D_2_O when using normal-sized ESI capillaries.

### Signal Suppression Depends on Analyte Acidity

Previous studies have reported a relationship between protein signal suppression, instrument polarity, and sodium adduction. Pan and McLuckey found that proteins with a high isoelectric point (pI) analyzed in negative ion mode, or proteins with a low pI analyzed in positive ion mode, exhibited reduced signal intensity accompanied by increased sodium adduction.^23^ We therefore next examined the effect of D_2_O on the electrospray ionization of different biomolecular systems with positive, neutral, or negative solution charges (Figure 3A, Table S1). First, we selected an engineered histidine-rich squid beak peptide devoid of acidic residues (HB*pep*, sequence GHGHY-GHGHY-GHGPY-K­(SP)-GHGPY-GHGHYW, where SP indicates connection to a disulfide-containing, self-immolative phenyl group).^24^ HB*pep* yields well-resolved negative ion spectra in H_2_O. In 90% D_2_O, we observed strong signal suppression and sodium adduction (Figure 3B). Next, we investigated the intrinsically disordered human protein tau (pI 8.2, predicted net charge at pH 7: +3), which showed complete signal suppression in 90% D_2_O in the negative ion mode similar to that of cytochrome *c*, despite being detectable in positive ion mode (Figure 3C). Since tau and cytochrome *c* are both basic proteins, we then extended the analysis to the acidic N-terminal (NT) domain from spider silk major ampullate spidroin 1 (pI 4.3, predicted net charge at pH 7: −3) which has been studied extensively by HDX-MS.^25,26^ Although signal intensity in negative mode was also in this case strongly suppressed in D_2_O compared to H_2_O, low-intensity signals could be detected in 90% D_2_O (Figure 3D), in contrast to cytochrome *c* and tau. This observation hence prompted us to include Acidic Nuclear Phosphoprotein 32 Family Member a (ANP32A), a partially disordered acidic protein with an extremely high negative charge density (pI 3.9, predicted net charge at pH 7: −61). Strikingly, this protein exhibited no obvious adduct-mediated deterioration by D_2_O in negative ion mode when compared to H_2_O (Figure 3G). Together, these data indicate that the adverse effects of D_2_O on protein electrospray ionization in negative mode are strongly dependent on protein acidity.

**Figure 3:**
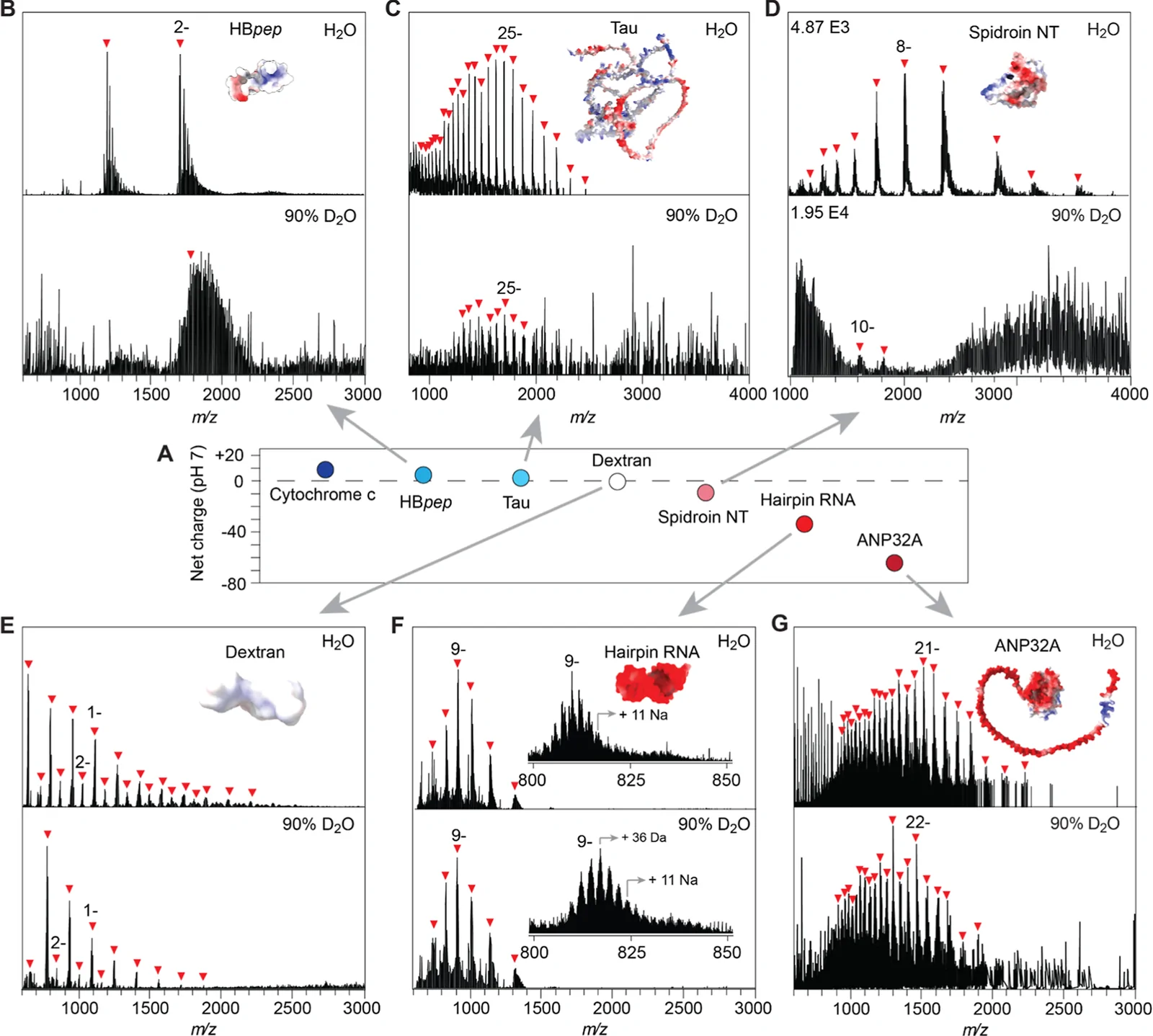
Determinants of D_2_O-induced protein signal suppression in negative ion mode. (A) Solution charges of the different biomolecules are shown in the central panel. (B) HBpep is readily detected in negative mode in H_2_O but exhibits extensive Na adduction and signal suppression in negative ion mode. (C) In negative ion mode, the mass spectra for disordered human tau protein show complete protein signal suppression in 90% D_2_O. (D) The partially negatively charged spidroin NT protein can be detected in negative ion mode mass spectra, but the signal intensity is notably reduced in D_2_O compared to H_2_O. (E) Negative ion mode mass spectra of dextran show deprotonation as the charge carrier. 90% D_2_O causes a mass shift of 3 Da per glucose unit with no additional effects. (F) Hairpin RNA exhibits a high number of charges and few sodium adducts in negative ion mode. 90% D_2_O induces a mass shift with no other notable effects. (G) Negative ion mode mass spectra of highly negatively charged ANP32A protein do not show a reduction in signal intensity in 90% D_2_O compared to H_2_O. The main charge states and corresponding sodium adducts are highlighted as insets. All molecules are shown as electrostatic surface renderings.

Having established protein acidity as a key determinant of D_2_O-induced protein signal suppression in negative ion mode, we next asked whether this effect extends to other classes of biopolymers with fundamentally different acid–base properties. Proteins are polyampholytes with moderate overall acidity, which usually supports ESI ionization in both positive and negative ion modes. In contrast, other biopolymers differ substantially: polynucleotides, such as RNA, are polyelectrolytes that are more acidic than proteins due to their phosphate backbone (p*K*_a_ ≈ 1), whereas neutral polysaccharides are considerably less acidic than proteins, as they contain only hydroxyl functional groups (p*K*_a_ ≈ 12).

We investigated the effect of D_2_O on the ionization of dextran, an α-1,6-linked glucose polymer (glucan). Mass spectra in positive ion mode using H_2_O as solvent were dominated by singly charged dextran chains, with a minor population of doubly charged species, both detected as sodiated adducts. In 90% D_2_O, each dextran chain exhibited a mass increase of approximately 3 Da per glucose unit, in agreement with the presence of 3 exchangeable hydroxyl groups per sugar residue, while the intensities of the doubly charged species were slightly reduced (Figure S3A). In negative ion mode, we detected singly deprotonated species without any sodium adducts (Figure 3E). As in positive ion mode, the presence of 90% D_2_O resulted in a 3 Da mass increase per glucose unit, with no notable differences in adduct formation or ionization behavior compared to H_2_O.

Mass spectra of the hairpin RNA (sequence GGCAGUGGCUUCGGCCGCUGCC) recorded in positive ion mode with H_2_O as solvent show a prominent 4+ charge state with up to 21 sodium adducts, in line with the presence of 21 phosphate groups in the backbone. The presence of 90% D_2_O in the ESI solution results in a mass shift of approximately 36 Da, as well as a slight shift toward the 5+ charge state and a minor reduction in the intensity of the sodium adduct peaks (Figure S3B). In negative ion mode, the RNA retains fewer sodium adducts (5–11) and a higher number of charges (6- to 10-), in line with the fact that the backbone phosphates carry the negative charge upon ionization (Figure 3F). Similar to the observations in positive ion mode, a mass shift of approximately 36 Da and a slight shift to higher charge states are detected in 90% D_2_O compared to H_2_O (Figures 3F, S3C). In summary, D_2_O does not cause adduct formation or ionization suppression for either polyanionic RNA or neutral polysaccharides in positive or negative ion mode.

### D_2_O Enables Selective Detection of an RNA Component in a Coacervate System

The observation that D_2_O affects the negative-mode ionization of different biomolecular classes to markedly different extents led us to ask whether electrospray ionization from D_2_O-containing solutions could bias mass spectra toward certain species over others when analyzing mixtures of biomolecules. For this purpose, we again turned to HB*pep*, which forms coacervates that encapsulate molecules such as RNAs for intracellular delivery at neutral pH (Figure 4A),^24,27^ making HB*pep*/RNA mixtures a relevant system for native and HDX-MS approaches. When the dilute phase of HB*pep* coacervates is analyzed under standard conditions, the MS signals from the peptide strongly dominate the mass spectra,^28^ potentially preventing the detection of other coacervate components such as RNA. Indeed, mass spectra in positive ion mode of HB*pep*-RNA coacervates show strong signals for the doubly and triply charged 3.6 kDa HB*pep* and none for RNA (Figure 4B). Addition of 90% D_2_O causes a shift to higher peptide charge states (Figure 4B). In negative ion mode, we detect monomeric HB*pep* with additional sodium adducts (Figure 4C). Strikingly, negative ion mode ESI in 90% D_2_O instead of H_2_O resulted in complete loss of the HB*pep* signal, whereas the RNA could be readily detected with well-resolved peaks, though with a slightly lower number of charges compared to RNA alone (Figures 4C, 3F). We speculate that the difference in the number of charges may stem from the weak interactions between HB*pep* and RNA that are disrupted during ESI. From these data, we conclude that D_2_O can selectively inhibit negative-mode ionization of polypeptides relative to RNA, leading to a pronounced polarity-dependent detection bias in mass spectra of phase-separated assemblies, making ESI-MS with D_2_O a suitable method for identifying components within mixed biomolecular systems.

**Figure 4:**
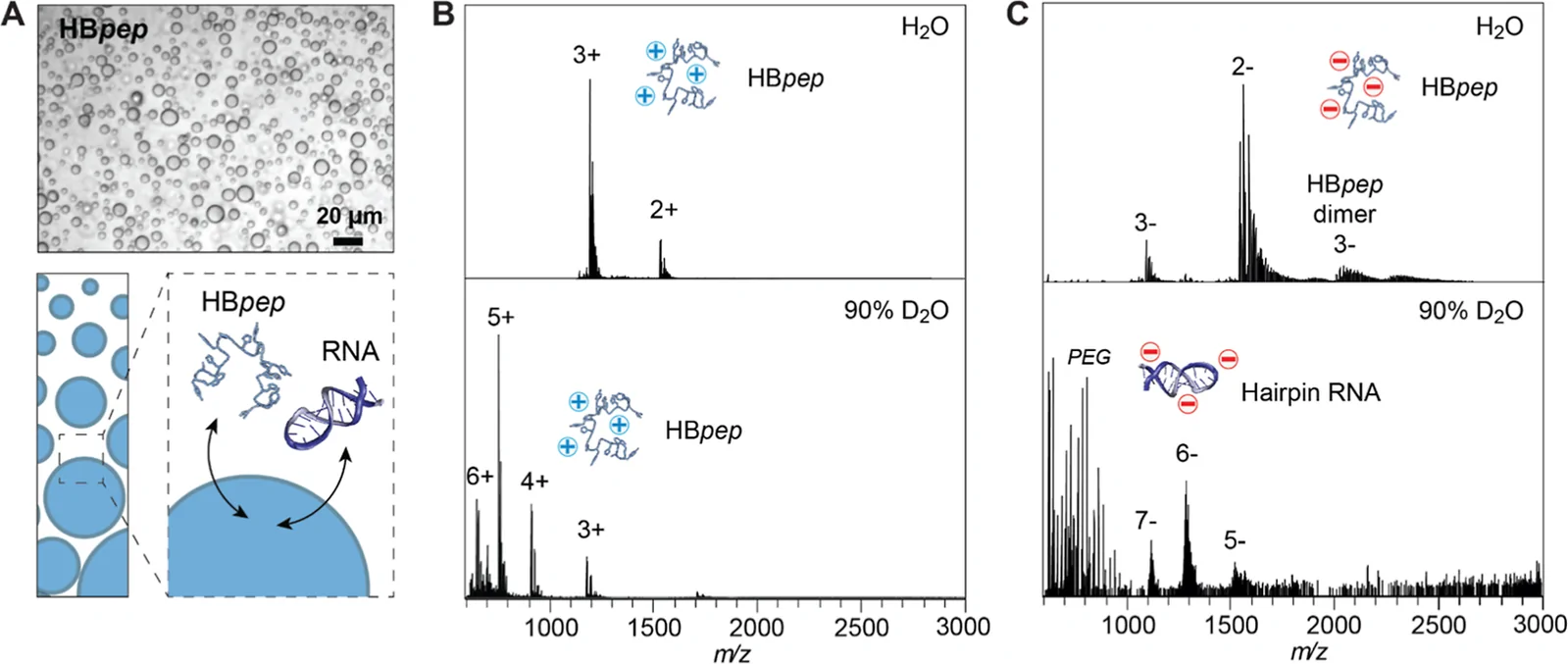
D_2_O enables selective RNA detection in negative ion mode. (A) HBpep forms micrometer-sized coacervates that can be visualized with brightfield microscopy and encapsulate cargo such as RNA. (B) Positive ion mode mass spectra of a 1:1 mixture of HBpep and RNA in H_2_O show preferential ionization of the peptide. 90% D_2_O causes an increase in the ESI charge of HBpep, but no other pronounced effects. (C) Negative ion mode mass spectra of a 1:1 mixture of HBpep and RNA in H_2_O show strong HBpep signals. In 90% D_2_O, peaks corresponding to RNA with reduced charge are detected, while HBpep signals are not observed. Low-intensity peaks indicating PEG contamination are visible in the low *m*/*z* region.

## Discussion

Here, we report that high D_2_O content causes extensive sodium adduction and signal suppression of positively charged proteins in negative mode ESI. This selectivity places strong constraints on the underlying mechanism, indicating that the observed effect does not arise from a general impairment of electrospray ion formation, but from an interplay between solvent-dependent droplet evolution and the intrinsic gas-phase charge stability of different classes of biomolecules.

In ESI-MS, nonvolatile adducts that are enriched in the droplet and attached to the analyte ions can cause signal suppression by extensive peak splitting.^29^ We observe a strong increase in sodium adduction with increasing D_2_O content. Sodium ions can attach to negatively charged side-chains as well as backbone carbonyls.^30^ Proteins are thus more vulnerable to adduct-mediated signal suppression than RNA, where the number of sodium adducts in negative ion mode is limited by the number of available backbone phosphates.^31^

We identify decreased S/N and adduct formation as potential causes of D_2_O-mediated protein signal suppression, both of which are more pronounced in negative than in positive ion mode. Strategies that reduce adduct formation, such as micrometer emitters that produce smaller droplets with fewer nonvolatile ions, collisional activation in the ion source to strip adducts, and the addition of ammonium acetate that competes for ion binding, all mitigate the protein ion suppression effect of D_2_O. Together, these findings support that D_2_O causes protein signal suppression primarily by promoting extensive sodium adduction in negative ion mode. Finally, McLuckey and colleagues reported that sodium ion incorporation was highest when protein charge in solution and ion charge in the gas phase were opposite.^23^ They propose that proteins with a solution charge that is opposite to ionization polarity are expelled from ESI droplets later than those where the solution charge matches the polarity. Being retained in the larger precursor droplets could expose the proteins to an increasing concentration of nonvolatile ions and increase sodium adduction.^23^ Their findings strongly support that a pronounced difference in sodium adduction is the driving force behind signal suppression in negative mode that is selective for basic proteins. Our results suggest that D_2_O can exacerbate this effect to the point of near-complete loss of protein signals in negative ion mode, where ionization is generally less efficient.

The observation raises the question of why D_2_O promotes sodium adduction in negative ion mode. H_2_O and D_2_O evaporate equally fast.^32^ D_2_O has a 25% higher viscosity,^12^ which is predicted to have a minute impact on droplet size.^33^ Furthermore, the greater strength of the O–D bonds compared to O–H implies that salts have a slightly lower solubility in D_2_O than in H_2_O, which may favor attachment to proteins.^34^ Negative ion mode promotes sodium adduction in general, likely due to the lack of protons that compete with cations for binding to negatively charged side chains. Ejections of negatively charged species have been observed in molecular dynamics simulations of positive ESI droplets, which may accelerate Rayleigh fission.^35^ Negative ESI droplets, on the other hand, remain further below the Rayleigh limit than positively charged droplets, likely reducing the efficiency of progeny droplet formation.^36^ None of these effects alone appear to be sufficient to explain our observations. However, the combination of less efficient sodium solubilization, less efficient removal of sodium ions from negatively charged droplets, and slightly larger droplet size, may elevate sodium adduction in negative ESI droplets to the point of complete protein signal suppression.

Beyond its mechanistic implications, the D_2_O-dependent bias can be exploited analytically. In HBpep/RNA coacervates, peptide signals dominate under standard ESI-MS conditions, potentially obscuring other components of the phase-separated assembly. In negative ion mode, replacing H_2_O with D_2_O selectively suppresses HBpep signals and enables RNA detection. Thus, D_2_O can act as a solvent-based selectivity switch that biases native mass spectra away from proteins and peptides and toward more highly anionic biomolecules. This effect should be considered when interpreting spectra from mixed biomolecular systems, but it may also provide a simple strategy for enhancing detection of nonprotein components in complex assemblies. Such an approach could complement structural techniques that exploit isotopic contrast, such as small-angle neutron scattering, where selective deuteration and contrast matching are routinely used to distinguish biomolecular components within complex assemblies.^37^

## Conclusions

Our results show that D_2_O is not an innocent solvent substitute in ESI-MS, but can introduce a pronounced polarity-dependent detection bias. In negative ion mode, D_2_O selectively suppresses many proteins and peptides, whereas highly anionic biomolecules remain readily detectable. This effect is associated with increased sodium adduction and altered droplet evolution, revealing an unexpected sensitivity of ESI to subtle solvent isotope effects.

These findings have practical implications for MS experiments that compare measurements in H_2_O and D_2_O, where changes in signal intensity may reflect ionization bias in addition to structural or exchange effects. At the same time, the effect can be exploited analytically, as demonstrated by selective RNA detection in peptide/RNA coacervates. Thus, D_2_O provides both a cautionary example and a useful tool for tuning biomolecular detection in native mass spectrometry.

## Materials and Methods

Deuterium oxide (99.9% D), horse heart cytochrome *c*, and dextran from *Leuconostoc mesenteroides* were purchased from Sigma. HB*pep* was synthesized and purified as described.^27^

Human full-length tau 411 was expressed in BL21 (DE3) *E. coli* cells induced for 2.5 h with 0.4 mM IPTG. Cells were harvested by centrifugation and resuspended in 50 mM NaP_i_, 1 mM EDTA pH 6.4 with 1*x* cOmplete protease inhibitor cocktail (Roche). Cells were disrupted by sonication (6 min, 2s on/8s off, 40%). Cell debris was removed by centrifugation and the supernatant was heat treated and centrifuged again to remove the insoluble fraction. After cation exchange, size exclusion chromatography (Superdex 200 PG 26/600) was used for further purification and the eluate was buffer exchanged to 20 mM NaPi, 0.2 mM EDTA, pH7.4.

ANP32A and NT were expressed in BL21 (DE3) *E. coli* cells by induction with 0.5 mM IPTG at an OD_600_ of 0.7–0.9 and incubated overnight at 20 °C. Cells were harvested by centrifugation, resuspended in 20 mM Tris–HCl, pH 8.0 supplemented with EDTA-free protease inhibitor cocktail (cOmplete, Roche), and lysed by sonication. After removal of cell debris by centrifugation, the cleared lysate was filtered and purified by immobilized metal affinity chromatography (IMAC). Eluted fractions were pooled and dialyzed at 4 °C overnight against 20 mM Tris–HCl, pH 8.0. Purified NT was concentrated to ∼1 mM and stored at – 20 °C. Purified ANP32A was supplemented with 1 mM DTT and subjected to TEV protease cleavage (1:100 w/w) overnight at 4 °C. Cleaved protein was separated from uncleaved protein and protease by reverse IMAC, dialyzed into 20 mM sodium phosphate buffer, pH 8.0, concentrated to ∼100 μM and stored at – 20 °C.

RNA was synthesized by in vitro transcription in 100 mM Tris-Glutamate pH 8 (Sigma-Aldrich), 15 mM Mg­(OAc)_2_ (Sigma-Aldrich), 7.5 mM Spermidine (Sigma-Aldrich), 10% DMSO (Sigma-Aldrich), 10 mM DTT (Sigma-Aldrich), 4 mM GMP (Sigma-Aldrich), 3 mM GTP, UTP, CTP (Thermofisher), 3 mM ATP (Sigma-Aldrich), 2 μM annealed dsDNA template (T7 promoter sequence TTAATACGACTCACTATA and template strand mGmGCAGCGGCCGAAGCCACTGCCTATAGTGAGTCGTATTAA, IDT) and 0.46 mg/mL T7 polymerase P266L (Protein Science Facility, Scilifelab).^39,40^ The reaction was incubated at 37 °C for 16 h at 300 rpm. RNA was purified by preparative 20% denaturing gel electrophoresis (C.B.S Scientific), eluted in 10 mM Tris–HCl pH 8.0 (Sigma-Aldrich), 1 mM EDTA (Sigma-Aldrich), and 300 μM NaCl (Sigma-Aldrich), then 0.2 μm sterile filtered (Whatman), and precipitated by first addition of 3x volumes glacial EtOH, and then incubated for 15 min in liquid nitrogen. After centrifuging at 16,000*g* for 30 min at 4 °C the desalted pellet was resuspended in 1 mL H_2_O and further buffer exchanged in 3 kDa Amicon centrifugal filters (Millipore) to an exchange factor of 1:10000. All proteins, RNA, and polysaccharides were prepared in deionized H_2_O to a final concentration of 20 μM and subjected to one round of desalting using ZebaSpin columns (Thermo Scientific).

### Mass Spectrometry

Standard metal-coated *n*ESI capillaries were purchased from Thermo Fisher Scientific and were used for all MS experiments unless otherwise noted. These capillaries were manually clipped open under a light microscope, typically yielding emitter opening-diameters of 10–15 μm.^22,41^ nESI capillaries with well-defined smaller diameters were pulled in-house using a Sutter P-1000 micropipette puller (Sutter Instrument, USA). These capillaries were pulled preopened, to emitter opening-diameters of approximately 1 and 4 μm, respectively.^21,22^ For polarity-switching experiments (Figure 1), the same emitter was used for both polarities.

Proteins were incubated in deuterium oxide for a minimum of 15 min, which corresponds to near-complete labeling of small proteins such as cytochrome *c*. Mass spectra were acquired on a Q Exactive Plus (Thermo Fisher Scientific), modified for high-mass analysis (MS Vision, NL). The capillary voltage was set to 1.5 kV, the source temperature to 30 °C and the fore vacuum (ion source) was set to 2 mbar. Sheath gas was nitrogen with a pressure of 0.1 bar. Orbitrap resolution was set to 70,000. All mass spectra from the QExactive were visualized using Xcalibur 2.2 (Thermo Fisher Scientific). For signal-to-noise quantification in cytochrome *c* spectra, four independent repeats were recorded for each condition and polarity. S/N ratios were calculated from 30 s of each acquisition, using the S/N tool in mMass 3.0 with the most intense protein peak as ref 42 Mass spectra with micrometer emitters were recorded on a Waters Synapt G2S (Waters Corp., USA). Ionization from nESI capillaries was initiated by applying a capillary voltage of 0.8–1.2 kV via a platinum wire inserted in contact with the sample solution. Source temperature was kept at 25 °C, sample cone voltage was set to 40 V, source offset voltage to 80 V and a trap voltage to 4 V. Gas flow settings were as follows: Trap cell 2 mL/min, He cell 180 mL/min, IMS cell 90 mL/min. All mass spectra from the Synapt were visualized using MassLynx 4.2 (Waters).

### Microscopy

For brightfield microscopy, 128 μM HB*pep* in Milli-Q was prepared in a 96-well half area low-binding plate (Corning) with transparent bottom and allowed to sediment for 1 min. Images were acquired using a Nikon Eclipse Ti series confocal microscope (Nikon Melville, NY, USA) equipped with an S Plan Fluor ELWD 40X DIC N1/0.6 NA (Nikon Melville, NY, USA), and a Zyla sCMOS camera (Andor, Oxford Instruments, Belfast, UK).

## Supplementary Material



## Notes

Supplementary data: deuterium uptake of cytochrome *c* in positive and negative ion mode; control mass spectrometry experiments in negative ion mode, cytochrome *c* in H_2_O with addition of sodium chloride or ammonium acetate; negative mode mass spectra of cytochrome *c* with intermediate sized (4 μm diameter) capillaries; positive ion mode spectra of dextran and RNA. Supplementary table: list of studied biomolecules with their predicted net charge at pH 7 (PDF)
