# Supplementary material for: An Isotope-Induced Polarity Bias in Electrospray Ionization Mass Spectrometry
Source: ACS Meas Sci Au. 2026 Jul 14;6(5):1636–44. doi: 10.1021/acsmeasuresciau.6c00134 (PMC13693268; doi:10.1021/acsmeasuresciau.6c00134)
Supplement: Supplementary file 1 [file tg6c00134_si_001.pdf]

Supplementary Information to

An Isotope-Induced Polarity Bias in Electrospray Ionization Mass Spectrometry

Qi Ying Neo, Henry T. P. Annecke, Irena Papageorgiou, Axel Leppert, Katja Petzold, Kevin Pagel, Ali Miserez, Erik G. Marklund, Nicklas Österlund, and Michael Landreh

[1] Department of Microbiology, Tumor and Cell Biology, Karolinska Institutet, 171 65 Solna, Sweden

[2] Center for Sustainable Materials (SusMat), School of Material Science and Engineering, Nanyang Technological University, 639798, Singapore, Singapore

[3] Department of Medical Biochemistry and Microbiology & Science for Life Laboratory & Center of Excellence for the Chemical Mechanisms of Life, Uppsala University, Uppsala Biomedical Centre, Uppsala 752 37, Sweden

[4] Department of Medical Biochemistry and Biophysics, Karolinska Institutet, 171 65 Stockholm, Sweden

[5] Department for Cell and Molecular Biology, Uppsala University, 751 24 Uppsala, Sweden

[6] Institute of Chemistry and Biochemistry, Freie Universität Berlin, 14195, Berlin, Germany

[7] Department of Molecular Physics, Fritz Haber Institute of the Max Planck Society, 14195, Berlin, Germany

[8] Department of Chemistry for Life Sciences, Uppsala University, 751 23 Uppsala, Sweden

* Correspondence to nicklas.osterlund@fu-berlin.de or michael.landreh@icm.uu.se

SI Figures 1-4 and Table 1

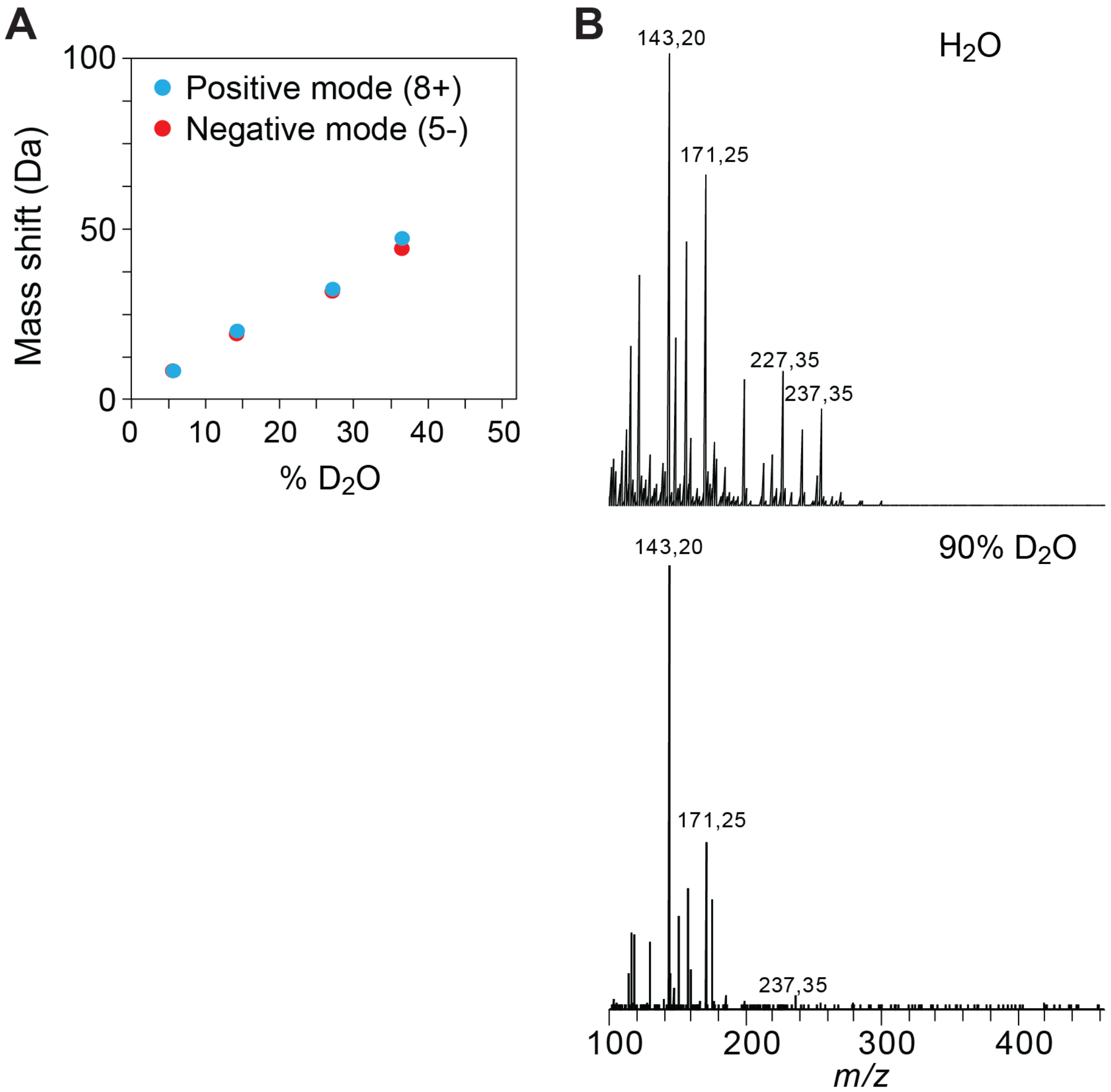

SI Figure 1. (A) Deuterium uptake of cytochrome *c* in positive and negative ion mode. No notable difference in deuteration as a function of D_2O content is observed. **(B)** The m/z region of mass spectra of cytochrome C in pure H_2O (top) and 90% D2O (bottom) show no indication of Na clusters or additional contaminants in the D_2O-containing sample.

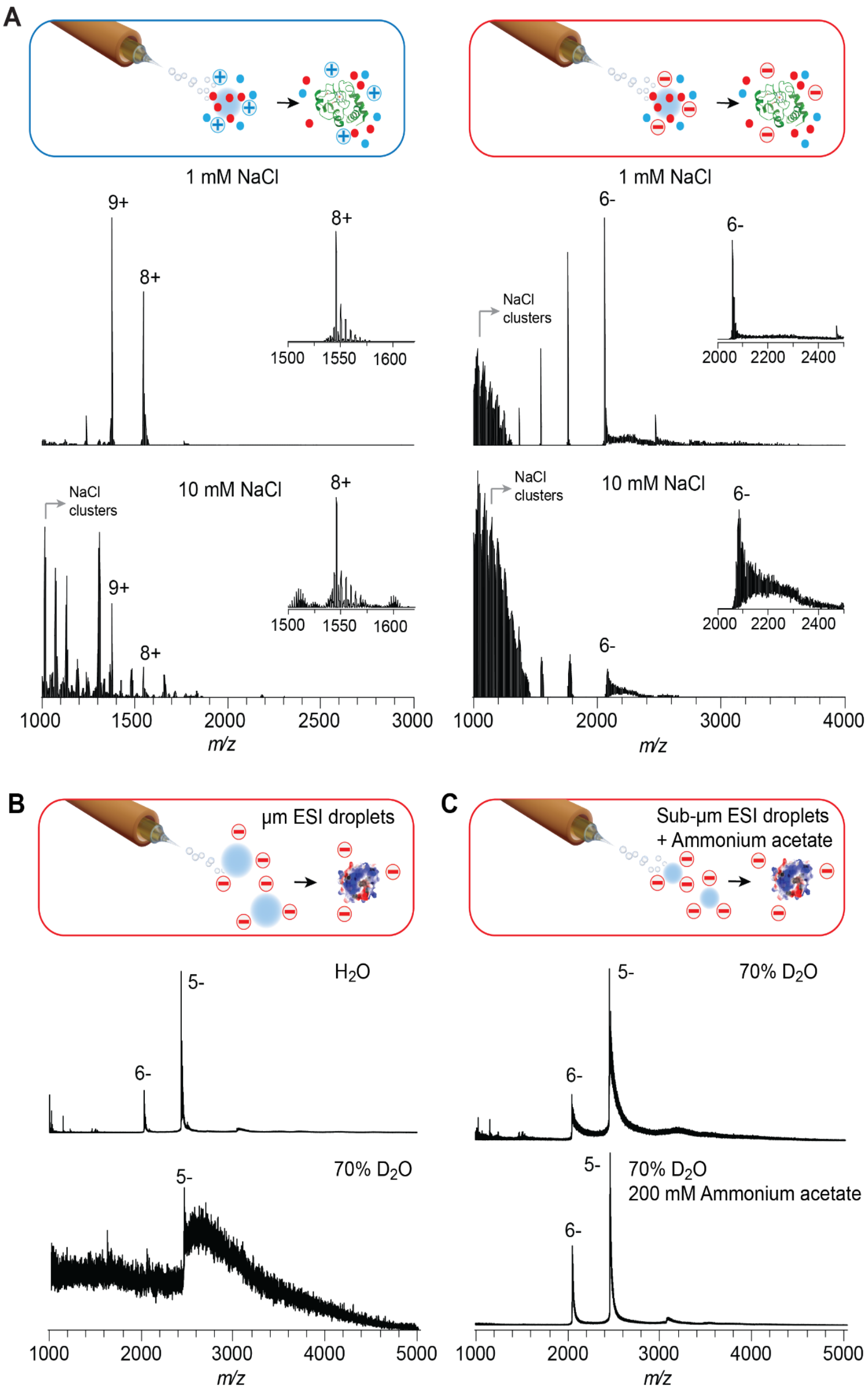

SI Figure 2. Signal suppression in negative ion mode is not caused by NaCl contamination but mitigated by modifying capillary orifice size or the addition of ammonium acetate. (A) Mass spectra of cytochrome c in H_2O containing 1 mM (top) or 10 mM (bottom) NaCl lead to appearance of clusters and notable adduct formation in both ionization modes but does not lead to complete signal suppression as seen for 90% D_2O. **(B)** Negative mode mass spectra of cytochrome c with 4 µm diameter capillaries show partial signal recovery and strong sodium adduction in 70% D_2O. **(C)** Negative-mode mass spectra of cytochrome c using 1 µm diameter capillaries show improved signal-to-noise and reduced sodium adduction with 200 mM ammonium acetate.

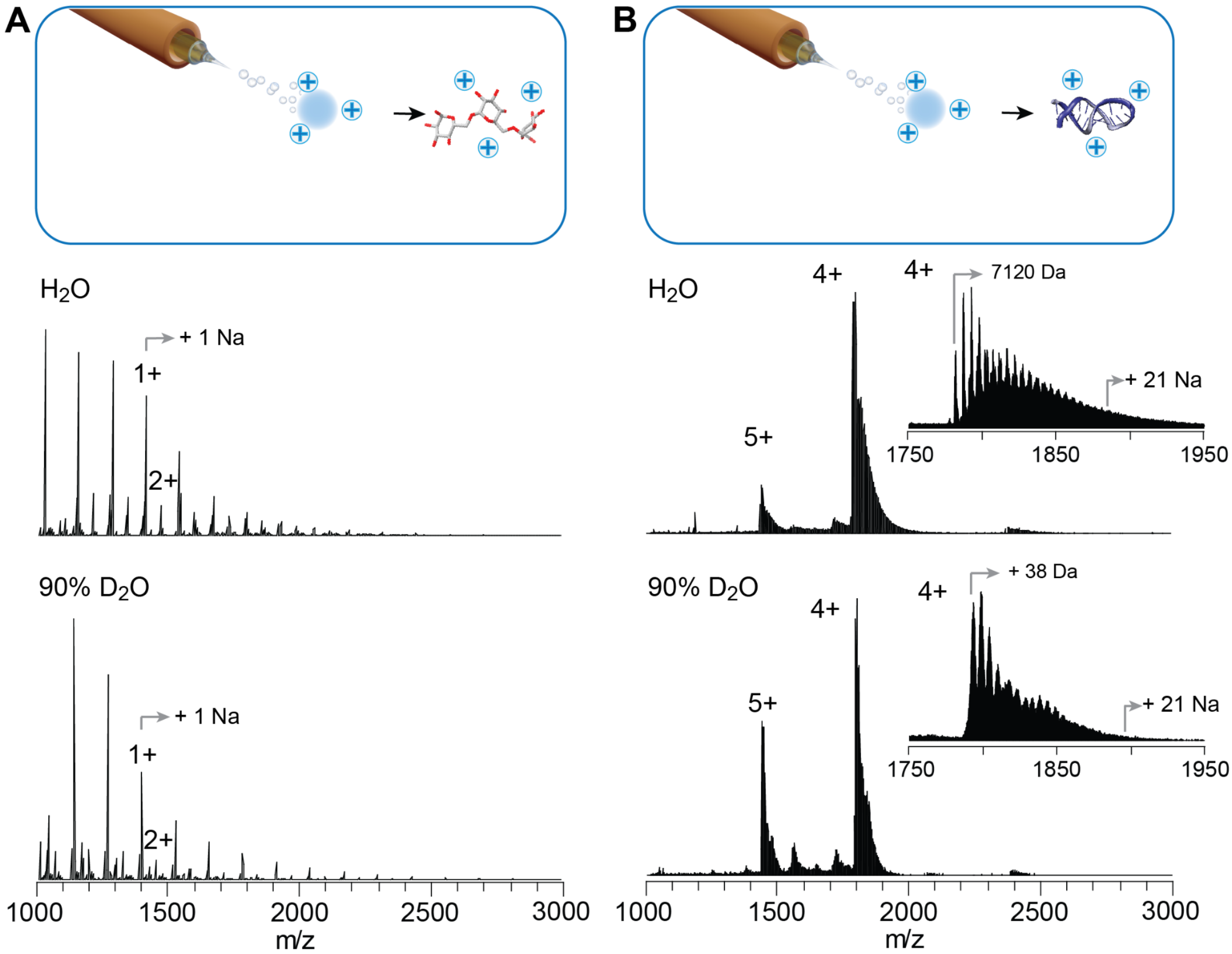

SI Figure 3. Positive ion mode spectra of dextran and RNA. (A) Mass spectra of dextran show a sodium adduct in positive ion mode. The addition of 90% D_2O causes a mass shift of 3 Da per glucose unit but no other effects. **(B)** In positive ion mode, RNA exhibits low charge and a narrow charge state distribution, as well as extensive sodium adduction to the backbone. Addition of 90% D_2O causes a mass shift of 38 Da but has no pronounced effect on ionization or adduct formation.

Table 1. Predicted net charge of various biomolecular systems at neutral pH and its suppression in D_2O in negative mode ESI.

Biomolecule	Biomolecular class	pI	Predicted net charge at pH 7	Signal suppression in D_2O
Cytochrome *c*	Protein	~10	+9.5	Yes
HB*pep*	Peptide	~8.4	+1	Yes
Tau	Protein	8.2	+3	Yes
Dextran	Polysaccharide	*n.a.*	0	No
Spidroin NT	Protein	4.3	-3	Yes
Hairpin RNA	Nucleic acid	< 3	-22	No
ANP32A	Protein	3.9	-61	No
